# Chronotropic incompetence in Chagas disease: usefulness of dual sensor pacemaker based on volume minute and accelerometer

**DOI:** 10.5935/1678-9741.20150011

**Published:** 2015

**Authors:** José Carlos Pachón

**Affiliations:** 1 International Board Heart Rhythm Examiners Certified Cardiac Device Specialist. Director of Pacemaker Service of Instituto Dante Pazzanese de Cardiologia (IDPC), São Paulo, SP, Brazil.

Chagas disease or American trypanosomiasis is a chronic parasitosis affecting most Latin American countries where an estimated 8 million people are infected. Current assessments have shown that even in the United States there is a total of 300,000 infected individuals, essentially all of whom are immigrants from the endemic countries. It is a tropical parasitic disease caused by the protozoan *Trypanosoma cruzi* that is transmitted to humans by blood-sucking triatomine bugs, known as "Kissing Bug", and via blood transfusion, mother-to-baby or organ transplant. This protozoan causes an acute systemic inflammatory illness that usually progresses to chronic myocarditis and autonomic disease leading to electrical and/or severe dilated cardiomyopathy. Myocardial damage is disseminated throughout the heart. The outcome may present cardiac arrhythmias, life-threatening heart failure, thromboembolism, stroke and sudden death. The latter is sometimes the first manifestation of the disease, even in the structurally normal heart (latent phase). In many patients, a chronic immunologic process may feed the inflammatory phenomenon even without the parasite. As a rule, extensive involvement of the autonomic nervous system and the cardiac conduction system results in a huge variety of supraventricular and ventricular cardiac arrhythmias. As a corollary, in approximately 60% of cases, the sinus node is injured, developing a more or less extensive sick sinus syndrome. Additionally, many patients have to be treated for heart failure and cardiac arrhythmias. Thanks to a coordinated multi-country program in the Southern Cone countries, the transmission of Chagas disease by vectors and via blood transfusion was interrupted in Uruguay in 1997, in Chile in 1999 and Brazil in 2006. For this reason, the incidence of new infections by *T. cruzi* across the South American continent has decreased by 70%. The natural course of the disease and the very common pharmacological requirement for treating numerous arrhythmias cause a high sick sinus node syndrome prevalence. One frequent expression of this condition is the "Chronotropic Incompetence" (CI).

READ ARTICLE ON PAGE 311

## Heart Rate Contribution to Exercise

Increasing heart rate during exercise is necessary for Chagas disease patients not only to adjust cardiac output but also to blunt the excessive sympathetic tone increase. The ability to perform physical work is enabled by a growth in oxygen uptake (VO_2_). During maximal aerobic exercise in a healthy person, VO_2_ increases approximately 4-fold, that may be composed of 2.2-fold increase in heart rate, 0.3-fold increase in stroke volume and a 1.5-fold increase in the arteriovenous oxygen difference. These data indicate that the heart rate increase is the most important contributor to the ability to perform sustained aerobic exercise. Therefore, it is well known that CI can be one important cause of a symptomatic exercise intolerance.

## Chronotropic Incompetence Definition

CI may be defined as the inability of the heart to increase its rate in proportion to physical activity or metabolic demand. Beyond Chagas disease, it is very common in many other cardiovascular illnesses and pharmacological interventions. It may produce exercise intolerance that causes symptoms and impairs quality of life. In addition, it is an independent predictor of major adverse cardiovascular events and overall mortality^[1,2]^. Nevertheless, the significance of CI is underappreciated, and it is often disregarded in clinical practice. In part, this may be due to multiple definitions, the confusing effects of aging, cardiopathy and medications, and the need for exercise testing for a conclusive diagnosis. In Chagas disease, CI has an additional risk as it may prone the patient to present a high and disproportional sympathetic tone during physical effort, as there is a deficient and delayed heart rate response. The association of high sympathetic tone with a marked electrical ventricular instability typical of these patients is one important additional sudden death risk. For this reason, sudden death occurrence during physical effort is well known in these cases. CI must be considered as potentially treatable, and its management can lead to a significant improvement in exercise tolerance, quality of life and complex arrhythmia risk reduction.

Technically, CI is considered when the heart rate fails to reach 85%, 80%, or, less commonly, 70% of the age-predicted maximum heart rate (APMHR), usually based on the 220 - age, obtained during a controlled incremental exercise test^[3]^ or failure to attain 80% of heart rate reserve (HR reserve= age-predicted maximal heart rate - resting HR) + resting HR^[4]^. Nevertheless, the lack of a consistent methodology and standardized criteria to determine CI probably justifies the high range of prevalence described in the literature, 9% to 89%, mainly in pacemaker patients^[5]^.

Despite being an exception as an isolated pacemaker indication, CI must be prevented and treated in all pacemaker's patients by programming the biosensor. The European Society of Cardiology pacemaker guidelines published in 2013 included CI as one isolated, very specific indication for pacemaker: "CI after heart transplantation: Cardiac pacing should be considered for CI impairing the quality of life late in the post-transplant period" (Class IIa, Level of Evidence C). In addition, they recommend that rate response features should be adopted in pacemaker patients with CI, especially if young and physically active, (Class IIa, Level of Evidence C)^[6]^. Despite being frequently underdiagnosed in clinical practice, this condition must deserve specific attention in Chagas disease patients with pacemakers, defibrillators^[7]^ or resynchronizators^[8]^, each of one with very peculiar considerations.

## Chronotropic Incompetence in Chagas Disease

As the sinus node is usually involved in the pathological process, this condition is very frequent in Chagas disease, even in the latent phase when the heart may be apparently normal. The inflammatory and fibrotic processes reduce the number of "P" cells, destroy the innervation and cause significant barriers to sinus node impulse progression. In addition, sera of patients with Chagas disease contain autoantibodies against both beta1 and beta2 adrenergic receptor subtypes with a theoretical gradual blockade of myocardial neurotransmitter receptors impairing both inotropic and chronotropic effects^[9]^. More recently, it has been shown that IgG fractions of sera from patients with chronic Chagas disease can decrease the HR of isolated rabbit hearts^[10]^. Patients in early stages (only serologic evidence of Chagas disease, but without segmental LV dysfunction) as well as those with baseline LV segmental wall motion abnormalities exhibit blunted myocardial contractile response to dobutamine infusion, CI and a biphasic inotropic response (i.e., similar to that seen in ischemic myocardium) to dobutamine despite the normal angiographic appearance of the coronary arteries^[11]^.

## Rate Response Pacemaker

When the sinus node is normal, it is the best sensor and the DDD pacemaker would not need a biosensor. However, even in this situation the biosensor is necessary to inform the system about physical activity and to manage the heart rate above the upper-track limit. The sleep-rate, for example, must be turned-off in case walking is detected. In addition, this resource may contribute to discriminating pathological and functional tachycardia in defibrillators.

When patients with complete AV block exercise, the sinus rate is significantly higher with VVI pacing than during dual chamber or VVIR modes, a response possibly reflecting the increased activity of the sympathetic nervous system when pacing is set to VVI mode^[12,13]^. This behavior strengthens the need for sensor programming and a good sensor tuning in Chagas disease patients, as the increased sympathetic tone is an additional risk factor for severe ventricular arrhythmias and sudden death in this pathology. Indeed, coronary sinus norepinephrine is higher in patients with VVI pacemaker during exercise. The increase in catecholamine on exercise during VVI pacing is likely to be related to the need for improving contractility and, consequently, cardiac output to compensate for the lack of rate response. This enhanced cardiac sympathetic activity may eventually produce an adverse effect on LV function, with the possible development of congestive heart failure and arrhythmias. On the other hand, an adequate chronotropic response that provides an appropriate increase in cardiac output during physical activity will surely reduce the risk of these arrhythmias. In addition, preliminary data suggest that rate adaptive AAIR and DDDR modes may be more efficacious in preventing atrial arrhythmias than their non-rate adaptive counterparts in Sick Sinus Syndrome^[14]^. DDDR pacemakers may prevent arrhythmias by eliminating the relative bradycardia noted during exercise in patients with non-adaptive devices, when excessive catecholamine release may increase the likelihood of atrial arrhythmias.

## Sensors for Rate Modulation

The most common are the sensor based on body mechanical vibration induced by movement and physical activity and the sensor that detects respiratory activity. Additionally, many other types of sensors have been tested and different parameters have been investigated to control the pacemaker rate: central venous temperature, changes of the right ventricular inotropism that may change impedance during the cardiac cycle (CLS, closed loop stimulation), QT interval, venous pH, oxygen saturation, stroke volume, minute ventilation, peak endocardial acceleration, gravitational sensor, etc.

There is a great range of advantages and disadvantages in these systems with respect to specificity and response proportionality, energy consumption, effort forecasting, ease of programming, auto-tuning, need for special leads, external interference susceptibility, influence of associated diseases and age limitation.

## Single sensor technology

The most used single sensor is based on a piezoelectric crystal or an accelerometer that senses mechanical activity. The first increases its electrical vibration in response to body mechanical activity, and the second is inertial and responds to the movement direction. Despite a quick response, it is not as specific as responding to vibrations related or not to physical activity and it has an inadequate response to isometric efforts and intellectual activity. In addition, its post-exercise frequency decay is not proportional to the duration of the effort. Nevertheless, its major advantages are simplicity and low power consumption.

The QT interval biosensor is based on the fact that this parameter shortens with the increase in metabolic activity, being almost always quite physiological. However, in the case of low T wave amplitude, it may have a reading error, it cannot be used in the atrium and it suffers interference of drugs that prolong or shortens the QT interval, as well as in pathological conditions such as ischemia and infarction, which can lead to an undesirable increase in heart rate.

The central venous blood temperature sensor is grounded on the premise that there is a temperature increase during physical effort related to muscle heat production and augmented circulation. However, in the early stages a drop in temperature of the central venous blood may occur, delaying the response. Moreover, it demands a particular lead that reduces their use, especially in cases of generator exchange when the patient has an implanted conventional lead.

The respiratory sensor is based on measurements of changes in thoracic bioimpedance as a result of changes in the quantity of air in the chest. It was improved by using the minute ventilation (MV) that takes into account both the respiratory rate and the tidal volume. It holds the advantage of using a conventional bipolar lead that can be implanted in the atrium or the ventricle. It is quite physiological given its high proportionality with the metabolic increase, independent of the quantity of mechanical action. Accordingly, it may change with mental activity and emotions. The post-exercise heart rate decay keeps great proportion to the intensity and duration of the activity performed. A disadvantage is the higher power consumption compared to the piezoelectric crystal sensor and the impossibility of using it in patients with lung diseases and pediatric age.

Several other sensors that rely on the need for special leads, such as those based on O_2_ saturation, pH, PEA (peak endocardial acceleration that means tip movement of the ventricular lead) have been less applied because of the complexity of the lead, the impossibility of using in generator exchange in patients with old leads and the non-applicability in the atrial chambers.

## Dual Sensor

Because none of the currently available sensors allow a response equal to the sinus node, there has been an effort of the cardiac pacing industry to associate two complementary sensors to yield a more physiological response. There are systems commercially available with the following associations: activity sensor and respiratory volume minute sensor (Medtronic, Boston and ELA Medical), activity sensor and QT sensor (Vitatron), activity and myocardial contractility sensor (Biotronik) and PEA with activity (Sorin). The combination most commonly used is the activity or accelerometer sensor with the respiratory volume minute sensor that brings the fast response of the first to the proportionality and specificity of the second resulting in a more physiological heart rate modulation. The goal is to harness the advantages and compensate for the shortcomings of each system^[15]^.

## Sensor in Chagas Disease

As CI is highly prevalent in this pathology, it is very important to program the pacemaker with this resource. Similar to the long QT syndrome, it would always be desirable that the sensor slightly anticipates the chronotropic needs of the patient by reducing sympathetic stimulation, since in these cases the catecholamines have an unwanted effect on the electrical stability of the myocardium. Especially in this condition, the well-adjusted sensor could have an effect enhancing the beta-blockers action by causing a reflex reduction in sympathetic tone. It can therefore be concluded that the dual sensor, allowing more fine-tuning and more physiologic heart rate, is highly desirable in this pathology. It is also highly recommended that in such cases a detailed program of the sensor be carried out, through exercise testing and/or Holter, in order to obtain the maximum arrhythmia suppression with proper adjustment of the heart rate response, while on the other hand, it is crucial to avoid ventricular dyssynchrony caused by excessive ventricular pacing due to a high biosensor driven heart rate. However, there are very few scientific studies addressing this issue. In addition, there are rare randomized studies comparing different frequency response modes in these patients. In this sense, it is appropriate to consider the results of the DUSISLOG study.

## DUSISLOG Study

The Dual Sensor *vs*. Single Sensor comparison using patient activity LOGbook (DUSISLOG) study was designed to find if there is any benefit from using dual-sensor for rate response in pacemaker patients. It is a two arms multicenter, prospective, randomized study enrolling 105 patients who received a rate-responsive pacemaker (Insignia, Guidant Corp.). After 1 month of DDD pacing at 60 ppm lower rate, a single sensor (Activity sensor or Minute Ventilation, randomized) was activated for 3 months at the manufacturer's nominal settings, followed by a 3-month period with dual sensors optimized for automatic response. During the last month of each period, the following data concerning patient physical activity were retrieved from pacemaker diagnostics Log: mean percentage of physical activity, mean intensity of activity. Quality of life (QoL) scores and 6-minute walk test (WT) were also recorded. A total of 105 patients achieved the enrollment criteria and completed all the specified follow-up program. WT, QoL, and patient activity significantly improved after activation of a single sensor and there was similar symptomatic benefit by using Activity or Minute Ventilation. However, no additional benefit was found using dual sensor. In spite of this, one important information came from the subgroup analysis. The patients were divided into three subgroups according to the degree of atrial CI, based on their percentage of sensed intrinsic atrial activity, calculated as % of atrial sense (AS) at 1 month follow-up (AS%=0, AS% <10, and AS% >10). A significant increase in the distance covered with the 6-mintue WT, QoL, and percentage of atrial pacing over 70 ppm was observed for single sensor, with an additional advantage of dual sensor, only in patients with major atrial chronotropic disease defined as the absence of intrinsic atrial activity over 60 bpm (0% AS%), which accounted for 17% of the enrolled population. Patients with moderate CI (0-10% AS%) showed only a trend toward symptomatic benefit from rate-responsive pacing, whereas those with mild or no CI (>10% AS%) had less or no benefit. Based on these data, it is worth to conclude that the rate responsive pacemaker with at least one sensor in Chagas disease is highly desirable, being the dual-sensor greatly recommended for the cases presenting severe CI.
